# Assessing the prevalence of psychotic symptoms in epileptic patients at a tertiary clinic

**DOI:** 10.4102/sajpsychiatry.v29i0.2062

**Published:** 2023-09-11

**Authors:** Michelle M. Hungwe, Karishma Lowton

**Affiliations:** 1 Department of Psychiatry, Faculty of Health Sciences, University of the Witwatersrand, Johannesburg, South Africa

**Keywords:** prevalence, psychosis, psychotic like experiences, epilepsy, ultra high risk for psychosis, prodromal questionnaire 16

## Abstract

**Background:**

The International League against Epilepsy (ILAE) defines epilepsy as a brain disorder characterised by an enduring risk to generate seizures with neurobiological, cognitive, psychological and social consequences. Psychotic disorders in epilepsy are a serious psychiatric complication affecting the prognosis, morbidity and mortality of patients. There is a paucity in literature with regard to the prevalence of psychotic symptoms in epileptic patients in low- to middle-income countries.

**Aim:**

This study aimed to look at the prevalence of psychotic symptoms in epileptic patients at an outpatient clinic using the prodromal questionnaire 16 (PQ-16).

**Setting:**

The study was conducted at the epilepsy clinic at Charlotte Maxeke Academic Hospital (CMJAH), a tertiary hospital located in Johannesburg, South Africa.

**Method:**

The PQ-16 was distributed to patients at the epilepsy clinic at CMJAH.

**Results:**

The study consisted of 121 participants. The prevalence of patients found to be at high risk of psychosis (i.e., PQ-16 score > 6) was 61.2% (95% lower confidence interval (LCI): 0.53, upper confidence interval (UCI): 0.70). None of the demographic variables showed significant associations in the percentage of patients found to be at high risk. No association was found between any antiepileptic drug and high risk of psychosis.

**Conclusion:**

The high prevalence of psychotic like experiences found suggests it is imperative to screen for psychotic disorders in epileptic patients and if required to involve neuropsychiatrists in their management.

**Contribution:**

This study highlights the importance of assessing psychotic symptoms in epileptic patients and the importance of a multidisciplinary approach in managing these complex patients.

## Introduction

The International League against Epilepsy (ILAE) defines epilepsy as a brain disorder characterised by an enduring predisposition to generate seizures and by the neurobiological, cognitive, psychological and social consequences of this condition.^1^ Approximately 50 million people worldwide have epilepsy, making it the most common neurological condition globally.^2^ It contributes 5% of the global burden of disease and has a significant impact on the economy.^3^ It is evident that epilepsy is a major global health concern and therefore every effort must be made to comprehensively treat epilepsy and all the associated aspects outlined in the definition from the ILAE. The terms ‘cognitive and psychological’ encompass the psychiatric disorders associated with epilepsy. Numerous studies have reported more psychiatric comorbidity in epilepsy than any other medical conditions.^1,4,5,6,7,8^ Salpekar et al. estimated that 20%–30% of patients with epilepsy (PWE) have some sort of psychiatric comorbidity ranging from anxiety and depression to neurocognitive disorders and psychosis.^5^ Psychosis is a constellation of symptoms ranging from positive symptoms, that is thought disorder, delusions and hallucinations to negative symptoms that is avolition and social withdrawal.^9^

There is a paucity of data with regard to rates of health service utilisation in PWE in Africa, but studies in the UK have shown that PWE with psychiatric comorbidity had higher rates of health service utilisation than PWE without psychiatric comorbidity.^10^ The World Health Organization (WHO) has acknowledged that epilepsy and psychiatric illness cannot be separated if epilepsy is to be adequately treated across the globe.^11^ As a result, WHO has piloted two programmes: the programme on reducing the epilepsy treatment gap and the mental health gap action programme. These two programmes aim to improve treatment and reduce the negative impacts of both epilepsy and psychiatric illness.^2^

Psychotic disorders are defined by the presence of two or more of the following abnormalities: delusions, hallucinations, disorganized thinking, grossly disorganized or abnormal motor behaviour and negative symptoms.^12^ Psychotic disorders in epilepsy are a serious complication affecting prognosis, morbidity and mortality.^9^ The lifetime prevalence of psychosis in the United Kingdom (UK) is 3%.^9^ The prevalence of psychosis in PWE ranges from 4% to 6% with a prevalence of up to 7% in populations with temporal lobe epilepsy.^2,4^ PWE are estimated to have an 8-fold increase in risk of developing psychosis.^13^

A recent study conducted in Thailand found a prevalence of psychotic disorders in PWE of 8.2%, which is higher than in the general population. They also explored the demographics of PWE and psychotic disorders and found no significant association between demographic characteristics and the presence of psychotic disorders in PWE.^6^

Multiple studies have explored the relationship between epilepsy and psychosis, with varying reports on the onset of psychotic symptoms in relation to the occurrence of seizures.^7,9^ These studies suggest that psychiatric disorders may be on a symptomatic spectrum of epilepsy. It is suggested that the psychiatric symptoms prior to seizure onset may be a neuropsychiatric prodrome phase or the epilepsy prior to psychiatric symptom onset may be a neurological prodrome to the psychiatric disorder.^14^

This hypothesis could have a massive impact on the treatment and diagnosis of both epilepsy and psychiatric disorders. The global understanding of the relationship between epilepsy and psychiatric disorders is vitally important as it informs the strategies to ensure adequate treatment with appropriate medication, appropriate usage of health resources and it helps to reduce stigma towards both epilepsy and psychiatric disorders and therefore improve the quality of life of patients.^13^

Psychosis in epilepsy can be described in many ways, namely interictal psychosis, post-ictal psychosis and psychosis secondary to antiepileptic drugs.^4^ Interictal psychosis is defined as any psychosis in clear consciousness that occurs in someone previously diagnosed with epilepsy and the psychosis is not exclusively during or immediately after the seizure.^15^ Interictal psychosis can resemble schizophrenia and pose a challenge to diagnostic clarity. However, there are some clinical features that can help distinguish one from the other such as negative symptoms present in schizophrenia, positive family history of schizophrenia and the episodic, fluctuating nature of interictal psychosis.

Postictal psychosis occurs when there is a clear temporal relationship between psychosis and the occurrence of a preceding seizure. This should not be confused with postictal confusion, which occurs immediately postseizure whereas with postictal psychosis there is a lucid interval between the seizure event and the onset of psychosis.^15^ A recent study by the neurology department in Vienna showed prevalence rates of interictal and postictal psychosis of 2.2% and 3.7%, respectively.^13^ There is a scarcity of data on the prevalence rates of interictal and postictal psychosis in South Africa. Most international studies on this topic are heavily reliant on video EEG monitoring, which is not readily available in South Africa.

A confounding factor in studies assessing psychosis in epilepsy is anti-epileptic medication and the link thereof. Prevalence rates have been described as 1%–2% with various contributions from the different agents (topiramate: 0.8%, vigabatrin: 2.5%, zonisamide: 1.9% to 2.3%, Levetiracetam: 0.3% to 0.7% and gabapentin: 0.5%).^2,9^ Determining whether the psychosis is because of the antiepileptic is difficult as omitting the drug as a causative agent of the psychosis may affect seizure control. A study was performed at the University of Melbourne investigating the clinical spectrum of antiepileptic drug-induced psychosis in PWE.^2^ Chen et al. showed that 1 in 7 patients with psychosis in epilepsy had an antiepileptic drug-induced psychotic disorder. A significant association between Levetiracetam use and the development of a psychotic disorder was noticed. Conversely, carbamazepine was found to have a negative association with a drug-induced psychotic disorder.^2^

To investigate the prevalence of psychotic symptoms, appropriate, easily administered screening tools can be helpful.^3,16,17^ Semi structured interviews such as the structured interview for prodromal symptoms (SIPS) and the comprehensive assessment of at-risk mental states (CAARMS) have been approved and validated for early detection of attenuated psychotic symptoms. Attenuated psychotic symptoms are symptoms that are below the threshold to qualify for a diagnosis of a psychotic disorder. They are less severe, more transient and the individual maintains reasonable insight into the psychotic-like experiences.^12^ The terms attenuated psychosis and psychotic like experiences are often used interchangeably. Unfortunately, these interviews are lengthy requiring a skilled clinician to administer them. The two most common screening tools used for attenuated symptom risk are the prodromal questionnaire and the prime screen revised.^17^ The Prodromal Questionnaire (PQ) is a 92 item, lengthy questionnaire, which was further condensed into the Prodromal Questionnaire-Brief (PQ-B) and Prodromal Questionnaire-16 (PQ-16).^3,18^

A French study recently showed that both neurologists and psychiatrists underrecognized and failed to treat epileptic psychoses.^15^ It is essential that psychiatry and neurology not only collaborate to treat epilepsy but also that neuropsychiatry takes a more prominent role in the treatment of epilepsy. Therefore, the aim of this study was to look at the prevalence of psychotic experiences of epileptic patients in an outpatient clinic using the PQ-16 and explore associations between antiepileptic medication, demographic factors, and the development of psychotic disorders in PWE by correlating these variables to the results of the PQ-16.

The study objectives were as follows:

- To determine the prevalence of psychotic symptoms among patients attending an epilepsy clinic in a tertiary hospital using the PQ-16
- To determine any associations between demographic factors and the occurrence of psychotic symptoms in PWE
- To determine any associations between antiepileptic drugs and psychotic symptoms in PWE
- To determine the temporal relationship between seizures and the onset of psychotic symptoms

## Research methods and design

### Study design and setting

This was a cross-sectional, quantitative study in which self-administered questionnaires were handed out to consenting participants. The study was conducted at the epilepsy clinic at Charlotte Maxeke Academic Hospital (CMJAH), a tertiary hospital located in Johannesburg, South Africa. Charlotte Maxeke Hospital services patients referred from the greater Johannesburg metro district.

### Participants

The patients seen at CMJAH are complex cases who were referred from primary or secondary level health care. The sample population included all consenting participants attending the epilepsy clinic at CMJAH who were over the age of 18, able to read and write in English and diagnosed with epilepsy. The questionnaires were handed out to all willing participants awaiting review with the neurologist. The participants were then given time to complete the questionnaire. The treating neurologists were asked to complete a section on the current antiepileptic drug treatment (AED) of the participant as well as the type of epilepsy the participant was diagnosed with. The questionnaires were then collected as the participants left the clinic after they had seen the neurologist. Patients with a pre-existing diagnosis of a psychotic disorder were excluded from the study. An assessment of *Z* scores indicated that a total sample size of 320 patients would detect significant differences at a low effect size. However, a sample size of 120 patients was adequate, assuming a large effect size.

### Instruments

The questionnaire used was the PQ-16, a validated self-report questionnaire used to screen for psychotic symptoms. The PQ-16 has good concurrent validity with the CAARMS and PQ-92.^16^ A score of six or more positive items on the PQ-16 showed 87% sensitivity and 87% specificity. The PQ-16 consists of 16 true or false questions addressing perceptual abnormalities, unusual thought content, paranoia and negative symptoms. It is recommended that the PQ-16 be used in conjunction with a more thorough clinical interview to conclusively determine whether a psychotic disorder is present or not.^16,17,18^ In this study, the PQ-16 will be used alone, purely for screening of psychotic symptoms and not for diagnostic purposes. An additional questionnaire containing demographics (age, race, level of education, marital status and employment status), medication, the type of epilepsy and the temporal relationship between seizures and psychotic symptoms was also administered. Permission to use the PQ-16 was obtained from the authors.

### Data collection

A total of 131 questionnaires were collected from November 2021 to May 2022. Five participants were excluded from statistical analysis because of incomplete questionnaires and incomplete consent forms. Four questionnaires were omitted from the results as they were completed by participants who had already submitted a questionnaire.

Some of the questionnaires had incomplete information about the current antiepileptic medication and the type of epilepsy. These participants files were collected and reviewed to complete the missing information.

### Statistical analysis

Statistical analyses were conducted in R software (version 4.00; www.R-project.org). The prevalence of ultra-high psychosis was calculated as a percentage of the total sample size with 95% CIs. The data set comprised of categorical data that were analysed using Pearson’s chi-squared goodness of fit, followed by binary post hoc tests for significant outcomes. Fisher’s exact tests for 2 × 2 and 2 × k data were used to test the associations between variables. In these analyses, categories of low scores from some variables were omitted from the analyses to increase the probability of detecting statistical significance. Tests were two-tailed, and the model significance was set at 0.05. Data are reported as counts and percentages and presented descriptively in tables, text or in a chart.

### Ethical considerations

The study was approved by the Wits Human Research Ethics committee – approval number M210404. Permission was obtained from the CEO of CMJAH and the head of department of neurology. Participation in the study was voluntary and informed consent was obtained from each participant. A distress protocol was included in the study.

## Results

The dataset comprised a total of 121 patients admitted to the epilepsy clinic at CMJAH. In reviewing demographic variables, statistical significance was found, fewer patients were > 60 years old (*p* = 0.009), were single (*p* < 0.001), had completed secondary school (*p* < 0.001) and were unemployed (*p* < 0.001) (Table 1). There were no significant differences in the number of female patients compared with male patients (*p* = 0.122) and the number of patients who received social grants compared with those who did not (*p* = 0.084) (Table 1). There were significantly more patients who had a diagnosis of unspecified epilepsy (*p* < 0.001) (Table 2). It was found that 66.1% of patients were taking more than one antiepileptic drug and significantly more patients were on lamotrigine and sodium valproate (*p* < 0.01). One patient was not taking any medication (Table 3).

**TABLE 1:** Socio-demographic characteristics of patients admitted to the epilepsy clinic at Charlotte Maxeke Academic Hospital.

Variables	Count	Percent	Statistics
**Age**			*p* = 0.009*
18–29	32	26.4	-
30–39	29	24.0	-
40–49	29	24.0	-
50–59	18	14.9	-
≥ 60	11	10.7	-
**Gender**			*p* = 0.122
Female	69	57.0	-
Male	52	43.0	-
**Marital status**			*p* < 0.001*
Single	89	73.6	-
Married	26	21.5	-
Widowed	5	4.1	-
Unknown	1	0.8	-
**Level of schooling**			*p* < 0.001*
Never schooled	7	5.8	-
Primary	12	9.9	-
Secondary	78	64.5	-
Tertiary	23	19.0	-
Unknown	1	0.8	-
**Employment status**			*p* < 0.001*
Employed	19	15.7	-
Unemployed	101	83.5	-
Unknown	1	0.8	-
**Social grant**			*p* = 0.084
Yes	70	57.9	-
No	51	42.1	-

* , *p*-value of < 0.001 is highly significant.

**TABLE 2:** The types of epilepsy in patients admitted to the epilepsy clinic at Charlotte Maxeke Academic. Hospital. Statistic = Pearson Chi-squared, which was significant.

Type	Count	Percent	Statistic
Adult-onset epilepsy	11	9.1	*p* < 0.001*
Atonic seizures	1	0.8	-
Childhood onset epilepsy	25	20.7	-
Complex partial epilepsy	1	0.8	-
Epilepsy- type not specified	58	47.9	-
Epilepsy due to brain tumour	1	0.8	-
Epilepsy due to meningioma	1	0.8	-
Generalised epilepsy	1	0.8	-
GTC epilepsy	10	8.3	-
Idiopathic epilepsy	1	0.8	-
Post infarct epilepsy	3	2.5	-
Post TBI epilepsy	5	4.1	-
Temporal lobe epilepsy	3	2.5	-

* , *p*-value of < 0.001 is highly significant.

GTC, generalised tonic clonic; TBI, traumatic brain injury.

**TABLE 3:** Anti-epileptic medication that was prescribed to patients admitted to the epilepsy clinic at Charlotte Maxeke Academic Hospital. Statistic = Pearson Chi-squared, which was significant.

Type	Count	Percent	Statistics
Carbamazepine	11	5.1	*p* < 0.001*
Lamotrigine	71	32.9	-
Levetiracetam	37	17.1	-
Phenytoin	1	0.5	-
Sodium valproate	71	32.9	-
Topiramate	17	7.9	-
Nil	1	0.5	-
Unknown	7	3.2	-

* , *p*-value of < 0.001 is highly significant.

### Objective 1

The prevalence of patients found to be at ultra-high risk (UHR) for psychosis (i.e. PQ-16 score > 6) among patients was 61.2% (95% LCI: 0.53, UCI: 0.70).

### Objective 2

**FIGURE 1:**
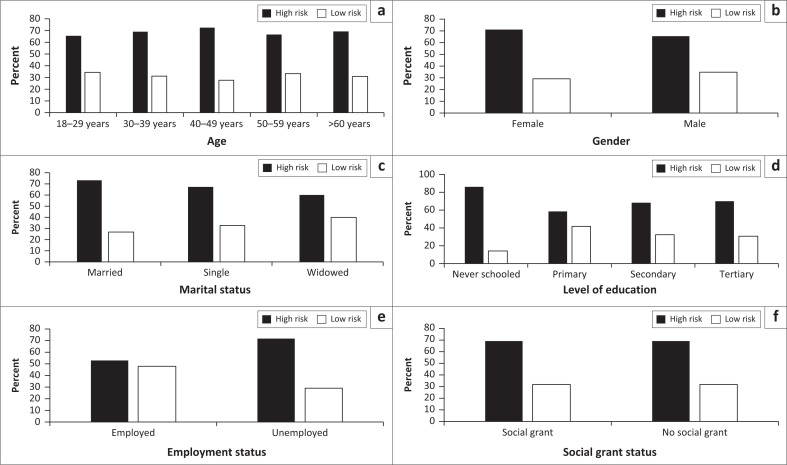
The percentage occurrence of ultra-high (PQ-16 score > 6) and low risk psychosis by socio-demographic factors for patients admitted to the epilepsy clinic at Charlotte Maxeke Academic Hospital.

None of the demographic variables showed significant variations in the percentage of patients was found to be high risk versus low risk psychosis, including age (Fisher’s exact test = 0.988), gender (Fisher’s exact test = 0.556), marital status (Fisher’s exact test = 0.755), level of schooling (Fisher’s exact test = 0.716), employment status (Fisher’s exact test = 0.117) and having a social grant (Fisher’s exact test = 1.000) (Figure 1).

### Objective 3

**FIGURE 2:**
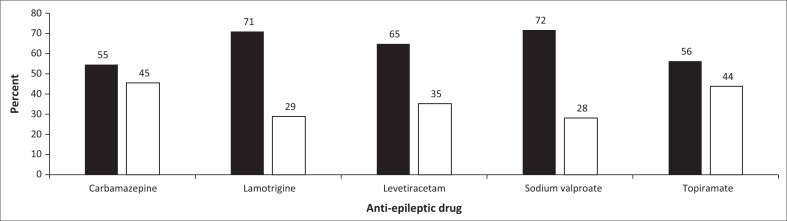
The percentage occurrence of ultra-high (PQ-16 score > 6) low risk psychosis by prescribed anti-epileptic medication in patients admitted to the epilepsy clinic at Charlotte Maxeke Academic Hospital.

No association between any specific antiepileptic drug and being at high risk of psychosis was found (Fisher’s exact test = 0.523) (Figure 2). The authors also compared Levetiracetam specifically with other antiepileptic drugs and being high risk of psychosis and found no significant difference (Fisher’s exact test = 0.192) (Table 4). Also found no significant difference between patients taking multiple antiepileptic drugs being found at high risk of psychosis compared with those taking one antiepileptic drug (Fisher’s exact test = 0.696) (Table 5).

**TABLE 4:** The comparison for Levetiracetam vs all the other AEDs. There is no significant difference (Fisher’s exact test = 0.192).

Drug type	High risk		Low risk	
	Count	Percent	Count	Percent
Levetiracetam	24	65	13	35
Other	115	69	52	31

**TABLE 5:** The comparison for 1, 2 and 3 drugs and high risk of psychosis. There is no significant difference (Fisher’s exact test = 0.696).

Number of drugs	High risk		Low risk	
	Count	Percent	Count	Percent
1 drug	29	70.73	12	29.27
2 drugs	36	75.00	12	25.00
3 drugs	13	54.17	11	45.83

## Discussion

### Key findings

#### Prevalence of ultra-high risk of psychosis

It was found that 61.2% of the patients attending the epilepsy clinic at CMJAH were at ultra-high risk for psychotic symptoms (i.e. had a PQ 16 score > 6). This is a much higher prevalence than in similar studies, which reported prevalence rates of 4%–6%.^2,4,6^ This difference may be accounted for by differences in the screening tool used and the parameters measured. Similar studies assessed for the prevalence of psychotic disorders fulfilling DSM criteria,^6^ whereas this study screened for psychotic symptoms and not diagnosable psychotic disorders. The prodromal questionnaire (PQ), from which the PQ-16 is derived, has been found to be less sensitive to the threshold between prodromal psychotic experiences and full psychosis.^3^ It is likely that the high prevalence reflects psychotic like experiences (PLEs) and not diagnosable psychotic disorders. Psychotic experiences occur commonly in the general population in the absence of a diagnosable psychotic disorder. Hanssen et al.^19^ found that 18.1% of adults in an unselected general population reported some type of psychotic experience in their lifetime and only 1.5% of them were diagnosed with a psychotic disorder.

The negative symptom subscale of the PQ has been noticed to have poor convergent and divergent validity. It has been shown that the factors assessing negative features have been more strongly correlated with depressive and anxiety symptoms.^20^ It is possible that the high prevalence rate is not only a reflection of PLE but also a reflection of anxiety and depression in sample population of this research. Sylla et al. conducted a study in Guinea and reported a prevalence of depression in PWE of 66.4%.^21^ A prevalence of 66.9% was found in a similar study in Benin and Togo.^21^ These prevalence rates from similar low-income countries are similar to the study’s prevalence of PQ16 scores greater than six and suggest the possibility of the results reflecting a questionnaire bias caused by PQ-16 detecting depressive symptoms.

The high prevalence rate may also have been affected indirectly by the COVID-19 pandemic. Psychotic symptoms are more likely to occur in patients with poorly controlled seizures^9^ and the pandemic negatively affected many epileptic patients’ seizure control. Recent studies have shown a direct correlation between the increased stress of the pandemic and increased seizures in PWE.^22^ It can therefore be extrapolated that an increase in the prevalence of psychotic symptoms would occur in response to the pandemic because of stress or even access to treatment during lockdown periods impacting adherence.

#### Socio-demographic factors

There was no association between high risk of psychosis and any socio-demographic factors. This is in keeping with a study performed in Thailand to assess the prevalence of psychiatric disorders in PWE.^6^ There is a paucity of literature on the socio-demographic factors influencing the development of psychosis in epilepsy, and no studies could be found to refute the findings of this study. There were significantly fewer patients in the sample of this study who were over the age of 60 years (*p* = 0.009); this could be a reflection of the lower average life expectancy of epileptic patients as opposed to the general population. The risk of premature death in epilepsy is up to three times greater than the general population.^11^ A significantly larger number of participants in the study’s sample were single (*p* < 0.001) was also noticed. A possible explanation could be related to the impact of refractory seizures and complications thereof on social and occupational functioning including relationships.

#### Antiepileptic medication

Antiepileptic drugs have been shown to cause psychotic symptoms in some PWE. Antiepileptic drug induced psychotic disorder (AIPD) has prevalence rates from 1% to 8.4% in clinical trials.^2^ Chen et al. found that Levetiracetam was the most common drug taken in patients with AIPD^2^ while Singh and Pandey showed that psychiatric morbidity increased in patients on multiple antiepileptic drugs.^5^ The study found no association between Levetiracetam or any particular antiepileptic drug and high risk of psychosis and no significant increase in risk of psychosis in patients on multiple antiepileptic drugs. The sample comprised complicated cases that required tertiary care and were mostly on multiple antiepileptic drugs. The researchers had very few patients on single antiepileptic drugs and could not make a meaningful statistical comparison. Multiple clinical trials have shown that Levetiracetam can cause irritability and aggression.^2^ Researchers found that significantly more of the participants were on sodium valproate and lamotrigine. The mood-stabilising properties of these agents could possibly counter the drug induced mood related side effects reported in previous studies.^2^

### Types of epilepsy

Qin et al.^23^ showed no difference in the risk of schizophrenia and schizophrenia-like psychosis between the different types of epilepsy. This is in keeping with the findings of this study, however both the results of this study and the study by Qin et al.^23^ are limited because two-thirds of the samples were unclassified types of epilepsy.

### Strengths and limitations

This study is to the best of the authors’ knowledge, the first of its kind to be carried out in an African setting. The WHO has highlighted that the prevalence of epilepsy is highest in low- and middle-income countries and the importance of fully treating the psychiatric comorbidity of epilepsy is becoming more apparent.^11^ This study sheds light on the high prevalence rates of psychotic symptoms in PWE in South Africa and thus provides motivation to improve psychiatric services available to PWE.

The PQ-16 was used to assess the presence of psychotic symptoms in epileptic patients. The PQ-16 is a validated tool used internationally with good reliability. It is also inexpensive, quick and easy to administer. This makes this study easy to replicate in other African settings.

This study was a cross-sectional study and therefore no inferences can be made about causation or progression from high risk of psychosis to diagnosable psychotic disorders. Researchers did not have a control group and thus could not compare the prevalence rate obtained with that of the general population in the given setting. The sample size was small and limited the power of the statistical calculations. The study sample consisted of patients with complicated and often treatment-resistant epilepsy and therefore the results cannot be generalised to the larger epileptic population. The PQ-16 was designed and validated in populations in which English is the first language of most participants. As a result of language differences, many patients may have found it difficult to understand some of the statements in the PQ-16. Many of the patients did not understand the Likert scale on symptom severity in the questionnaire and thus did not complete the section. As a result of the low numbers of responses to the severity grading, it was excluded from the final results. The severity scale has been noticed to improve specificity of the prodromal questionnaire brief: a predecessor to the PQ-16.^9^ Without the severity scale, the authors were unable to obtain a prevalence rate that reflects purely psychosis. Despite the various limitations, this study highlights the high prevalence rate of psychotic-like experiences in PWE in this particular population and provides valuable information to assist us with improving the quality of life of these patients.

## Conclusion

Findings show a high prevalence of psychotic-like experiences in PWE attending a tertiary epileptic clinic. This finding shows how imperative it is to not only screen for affective disorders in epileptic patients but also to screen for psychotic disorders. It also suggests the need for further involvement of neuropsychiatrists in the treatment and management of epilepsy.
